# Medication prescribing errors in the medical intensive care unit of Tikur Anbessa Specialized Hospital, Addis Ababa, Ethiopia

**DOI:** 10.1186/s13104-015-1435-y

**Published:** 2015-09-16

**Authors:** Oumer Sada, Addisu Melkie, Workineh Shibeshi

**Affiliations:** Department of Pharmacy, College of Medicine and Health Sciences, Wollo University, Dessie, Ethiopia; Department of Internal Medicine, School of Medicine, Addis Ababa University, Addis Ababa, Ethiopia; Department of Pharmacology and Clinical Pharmacy, School of Pharmacy, Addis Ababa University, Addis Ababa, Ethiopia

**Keywords:** Medication error, Prescribing error, Intensive care unit

## Abstract

**Background:**

Medication errors (MEs) are important problems in all hospitalized populations, especially in intensive care unit (ICU). Little is known about the prevalence of medication prescribing errors in the ICU of hospitals in Ethiopia. The aim of this study was to assess medication prescribing errors in the ICU of Tikur Anbessa Specialized Hospital using retrospective cross-sectional analysis of patient cards and medication charts.

**Results:**

About 220 patient charts were reviewed with a total of 1311 patient-days, and 882 prescription episodes. 359 MEs were detected; with prevalence of 40 per 100 orders. Common prescribing errors were omission errors 154 (42.89 %), 101 (28.13 %) wrong combination, 48 (13.37 %) wrong abbreviation, 30 (8.36 %) wrong dose, wrong frequency 18 (5.01 %) and wrong indications 8 (2.23 %).

**Conclusions:**

The present study shows that medication errors are common in medical ICU of Tikur Anbessa Specialized Hospital. These results suggest future targets of prevention strategies to reduce the rate of medication error.

## Background

Pharmaceutical care is the responsible provision of drug therapy for the purpose of achieving definite outcomes that improve patients’ quality of life. Any suboptimum therapy leads to medication error [1]. Medication errors (MEs) are defined as any preventable event that may cause or lead to an inappropriate medication usage, or harms the patient, while in the control of the health care professional, patient or consumer. Errors in the medication process can occur at different stages: prescribing, transcribing, dispensing and administration [2].

Medication errors are one of the most common types of medical errors and one of the most common and preventable causes of iatrogenic injuries [3]. These events may occur due to professional practice, health care products, and procedures such as prescribing, dispensing, and administration. High error rates with serious consequences are most likely to occur in intensive care units (ICUs) but errors are minimized in the presence of intensivists [4, 5]. Various studies have been carried out to find out the impact of MEs; but the issue received maximum attention in the immediate years after the Institute of Medicine (IOM) report 1999 was published [1]. This was followed with considerable interest by the medical community. However, to date, there is little evidence that patient safety has improved [6]. In general, some units such as ICUs are more prone to errors, because of complex settings and medical conditions of patients [2].

Prescribing errors in critical care units are frequent, serious, and expected, since these patients are prescribed twice as many medications as patients outside critical care [5]. In the ICU, on average, patients experience 1.7 errors per day and nearly all suffer a potentially life threatening error at some point during their stay than patients in other hospital wards due to their decreased physiological reserves which increase the risks of harm from medication-related errors. However, there is wide variation in the definition of errors and the methods used to detect them [4, 5, 7]. MEs account for 78 % of serious medical errors in the ICU [6]. Factors contributing to the frequency of MEs include: unobtainable medical history, since most patients in the ICU are sedated, multiple medications being received, most medications in the ICU being given intravenously, where calculation of infusion rates is often required; and insufficient staff numbers [4, 5, 8]. For these reasons, literature has long supported the concept of ‘pharmacist participation’ in the prescribing stage of medication orders, aiming to reduce the number of prescribing errors [9, 10]. Few epidemiological data are available regarding the prevalence, type and causes of MEs in ICUs of developing countries including Ethiopia. This study focused on prescribing errors because, prescription is hand written and susceptible to error during prescription writing process and the absence of any system to support prescribing physicians, who usually rely on their memory, to ensure correct prescribing practice. The objective of study was to assess medication prescribing errors rate in the ICU Tikur Anbessa Specialized Hospital (TASH), a tertiary care teaching hospital found in Addis Ababa, Ethiopia.

## Methods

### Study area

This study was conducted in the medical ICU of TASH. It is the largest specialized hospital in Ethiopia, provides a tertiary level referral treatment, with over 700 beds, and serves as the training center for undergraduate and postgraduate medical students, dentists, nurses, midwives, pharmacists, medical laboratory technologists, radiology technologists, and others who shoulder the health problems of the community and the country at large. The medical ICU has six beds and serves critically ill patients from different departments of the hospital.

### Study design

A retrospective cross-sectional study was conducted from April 15 to May 15, 2013 by using charts of patients who were admitted in the medical ICU of TASH in the 1 year period (March 1, 2012 to March 30, 2013).

### Inclusion and exclusion criteria

All medication-prescribing interventions by all prescribing physicians for patients admitted to the medical ICU of TASH during the 1 year period were included in the study. Patients with age >12, irrespective of diagnosis, and gender all who were admitted in the medical ICU in the 1 year period and given medication were included. Patient cards and medication charts with missed variables, incomplete information, lost cards and with no medication order were excluded from the study.

### Sample size determination

The sample size was calculated using single proportion formula and adjusted sample size was finally calculated using a prevalence value of 0.52 from previous study which results in sample size of 220. The sampling frame was obtained from patient registration form, which is all patients who were admitted to the medical ICU of TASH in the last 1 year period, and then random sampling technique was used to select subjects until 220 charts were obtained.

### Study variables

Prescribing error was the dependent variable and co-morbidities, age, state of the patient at admission, poly-pharmacy, length of ICU stay, route of administration, experience of prescriber were independent variables.

### Data collection and analysis

Data were collected, using a structured format, by two pharmacy practice postgraduate students who were trained on how to obtain data from patient cards and medication charts. The content includes demographic variables, dates and times of prescription, name of the medications, dosage forms, doses and frequency of medications prescribed. The clinical findings, the laboratory and diagnostic results were used to determine appropriate medications. Prescribing errors that could be identified with chart review alone were determined by comparing prescribed drugs with up-to-date standard treatment guidelines, textbooks, and software [11–14].

Data were edited, coded, and entered into SPSS (Windows v 16.0). Descriptive statistics was computed to determine the overall prevalence of prescribing errors and logistic regression was generated to determine risk factors. The results were expressed as mean ± standard deviation, median and/or percentage as appropriate. The p values less than 0.05 were considered significant.

### Potential clinical consequences

A four-scale unambiguous classification system was developed including the following categories—potentially fatal, potentially serious, potentially significant, and potentially non-significant. Definitions of potentially fatal and potential serious errors were in accordance with international definitions of potential (ADEs) [15, 16]. The principal investigator and one internist classified these errors in these four categories.

### Operational definitions

*Prescribing error* Implies deviation of medication prescribing from standard practices (as indicated in standard treatment guidelines, textbooks, and software) excluding, indication without drug, dosage form errors, illegible hand writing, and failure to authenticate the prescription with signature and/or date.

### Poly-pharmacy: the use of five or more regular medications at a time

*Wrong combination* Implies therapeutic duplications and clinically significant drug interactions.

*Omission error* Implies medications ordered without specifying dose, frequency, and route.

*Wrong indication* Implies the presence of inappropriate drug and contraindications which were not noted by the prescribing physician.

### Ethical considerations

This study was approved by Ethical review board of School of pharmacy, Addis Ababa University (ERB/SOP/28/05/2013). The collected data was used exclusively by the researchers, guaranteeing the privacy of the information obtained.

## Results

### Characteristics of participants

This study included 882 physician drug prescription episodes for 220 patients who were admitted to the medical ICU of TASH during the 1 year period in 1311 patient-days. Several prescriptions did not contain information such as drug allergy history and/or weight of patient and were excluded from definition of prescribing error.

Out of 220 patient cards, 120 (54.5 %) were male patients. The age profile showed that only 13 (5.91 %) patients were below the age of 18 years; 136 patients (61.8 %) were between 18 and 50 years and 71 (32.27 %) patients were over 50 years of age and the mean age of 41.34 (±17.7) years. 168 (76.36 %) of them were admitted to medical ICU directly from emergency outpatient department (EOPD). 161 (73.2 %) of the patients were conscious and 187 (85 %) were having a prescription with poly-pharmacy, with the average number of medications per patient being 8.8 (±3.81) drugs. The median length of stay in the ICU was 5 (1–27) days until death or transfer to other wards, and the average diagnosis per patient was 3.29 (±.44) (Table 1).

**Table 1:** Epidemiological and clinical characteristics of the study patients at Tikur Anbessa Specialized Hospital, Addis Ababa, Ethiopia (n = 220)

Characteristics	Frequency (%)
*Age*	
<18	13 (5.9 %)
18–50	136 (61.82 %)
>50	71 (32.27 %)
*Sex*	
Male	120 (54.5 %)
Female	100 (45.5 %)
*State of patient*	
Conscious	161 (73.2 %)
Unconscious	59 (26.8 %)
*Regimen taken*	
Poly-pharmacy	187 (85 %)
Simple	33 (15 %)
*Source of admission*	
Emergency^a^	168 (76.36 %)
Other ward	52 (23.64 %)
*Median length of stay in ICU*	5 (1–27) days
*The average no. of Dx/patient*	3.29 (+1.44)

*Dx* diagnosis

^a^A patient was directly admitted to ICU from emergency

### Identification of medication-related events

Of the 882 prescription episodes, approximately 26.42 % orders had at least one medication error. The majority of orders were having one error per order which occurred in 148 (16.78 %) orders, followed by two errors per order 44 (4.9 %), three errors per order occurs in 31 (3.51 %), four errors in 5 (0.57 %), five errors in 2 (0.23 %) orders. The total number of errors identified was found to be 359, which accounts error prevalence of 40.7/100 orders. The prescribing errors were classified according to the types of errors and the medication class involved.

Out of the total errors detected, omission errors were noted 154 (42.89 %) of the total errors identified making it the top most medication error. This was followed by wrong combination in 101 (28.13 %) cases, 48 (13.37) % wrong abbreviation. The detailed distribution of the medication errors is shown in Table 2.

**Table 2:** Frequency (%) of medication prescription error categories in Tikur Anbessa Specialized Hospital, Addis Ababa, Ethiopia

Error type	Frequency (%)
Dose omission	33 (9.19 %)
Frequency omission	58 (16.15 %)
Route omission	63 (17.54 %)
Omission error (subtotal)	154 (42.89 %)
Drug interaction	57 (15.87 %)
Duplication	44 (12.26 %)
Wrong combination (subtotal)	101 (28.13 %)
Wrong abbreviation	48 (13.37 %)
Over dose	14 (3.89 %)
Under dose	16 (4.46 %)
Wrong dose (subtotal)	30 (8.36 %)
Wrong frequency	18 (5.01 %)
Wrong indication	8 (2.23 %)
Total	359 (100 %)

The analysis of medication errors identified per medication class showed that the cardiovascular agents contributing maximum (33.9 %), this was followed by antimicrobial agents (20.49 %). Approximately 80 % of the total errors occurred in cardiovascular, antimicrobial and gastrointestinal agents (Table 3).

**Table 3:** Therapeutic category of medications with prescribing errors at Tikur Anbessa Specialized Hospital, Addis Ababa, Ethiopia

Drug category	Frequency (%)
Cardiovascular drugs	139 (33.90 %)
Antimicrobials	84 (20.49 %)
GI drugs	77 (18.78 %)
Opioid	34 (8.29 %)
CNS drugs	33 (8.05 %)
Analgesic and sedatives	21 (5.12 %)
Miscellaneous^a^	22 (5.37 %)
Total	410 (100 %)

^a^Bronchodilators, minerals, vitamins, drugs of autonomic nervous system, electrolytes

It is evident that the total number of times a medication class was involved in the errors (410) was higher than the total number of errors identified (359). This implies that in one error, two or more medications might be involved belonging to either the same or different class (Table 3).

Sixty-nine drugs were related to prescribing errors. The top five drugs with high incidence of medication error included; cimetidine 48 (11.71 %) of which 31 (7.56 %) were drug interactions and 17 (4.15 %) were duplication errors. Followed by clopidogrel with an error rate of 41 (10 %), in which all of them were drug interactions. In the third rank was Omeprazole 20 (4.88 %) of which 19 (4.63 %) were drug interactions and 1 (0.24 %) was duplication error, followed by dopamine 19 (4.63 %), 18 (4.39 %) of them were omission errors and only 1 (0.24 %) was dose error and tramadol with error rate of 18 (4.39 %), 16 (3.9 %) of them were duplication errors and 2 (0.49 %) were wrong dose errors. More than one-third of the total error occurred in these five drugs (35.6 %).

The evaluation of potential consequences of prescribing errors indicated that the majority of the errors were non-significant (67.4 %) and no fatal error was detected (Table 4).

**Table 4:** Potential clinical consequences of prescribing errors detected at Tikur Anbessa Specialized Hospital, Addis Ababa, Ethiopia

Clinical consequences	Frequency (%)
Potentially fatal	–
Potentially serious	6 (1.7 %)
Potentially significant	111 (30.9 %)
Potentially non-significant	242 (67.4 %)
Total	359 (100 %)

### Factors associated with prescribing errors

The binary logistic regression analysis showed that there was statistically significant association between prescribing error and age >50 years (p = 0.003) and 18–50 years (p = 0.004). The odds of these age groups [OR = 10.771 (2.208–52.546) and 9.765 (2.079–45.861)] respectively revealed that the probability of error in the age group >50 years is 10.771 times higher than patients with age <18 years. Poly-pharmacy was also significantly associated with error rate with (p = 0.000) and odds ratio [OR = 5.644 (2.473–12.884)].

The error rate in patients getting poly-pharmacy was 5.644 times higher than in patients getting simple regimen. The odds of both age and poly-pharmacy were large enough (>3) indicating that there was strong association with error rate. Male gender (p = 0.019) and admission from other wards (p = 0.029) were also found to be significantly associated with error rate. The odds of these factors were (OR = 1.553 and 1.889) respectively showed there was moderate association with error rate. Whereas, state of patient at admission, duration of ICU stay and number of co-morbidities were not significantly associated with prescribing error (Table 5).

**Table 5:** Frequency (%) of medication prescription errors according to potential risk factors at Tikur Anbessa Specialized Hospital, Addis Ababa, Ethiopia

Characteristics	Number of patients	No. of patients with error	% of patients with error	P-value	Ex(B) (95 % CI)
Age					
<18	13	2	15.4	–	
18–50	136	87	64.0	0.004	9.765 (2.079–45.861)
>50	71	47	66.2	0.003	10.771 (2.208–52.546)
Gender					
Male	120	73	60.8	0.019	1.553
Female	100	63	63.0	–	1.096 (0.634–2.124)
Regimen taken					
Poly pharmacy	187	127	67.6	0.000	5.644 (2.473–12.884)
Simple	33	9	27.3	–	0.375
Co-morbidity					
<3	75	41	54.7	–	1.206
≥3	145	95	65.5	0.117	1.576 (0.892–2.784)
State of patient					
Conscious	161	101	62.7	–	1.458
Unconscious	59	35	59.3	0.645	1.154 (0.627–1.894)
Source of admission					
Emergency	168	102	60.7	–	0.818 (0.427–1.567)
Other wards	52	34	65.4	0.029	1.889
Length of ICU stay					
<4 days	79	43	54.4	–	1.194
≥4 days	141	93	66.0	0.092	1.622 (0.923–2.849)

Sample examples of different types of prescribing errors (omission, wrong combination, wrong dose, frequency and wrong indication) detected in the present study are indicated in Table 6.

**Table 6:** Examples of medication prescribing errors at Tikur Anbessa Specialized Hospital, Addis Ababa, Ethiopia

Faulty use of medications detected
Tramadol 50 mg IV TID was prescribed with morphine 30 mg po BID and pethidine 50 mg IV TID where one/two of these drugs is enough (duplication error)
Nimodipine was initially prescribed on three times daily(TID) basis instead of Q4 h (wrong frequency)
A patient with peptic ulcer disease (PUD) was initially taking esomeprazole 20 mg IV bid was given cimetidine 200 mg IV bid and ranitidine 50 mg IV bid where one drug is enough (duplication error)
Tramadol 500 mg IV TID was prescribed for 66 years old patient to control pain, instead of 50 mg IV TID (wrong dose)
Forgot to define the route of administration of heparin as IV or SC (omission error) Forgot to define the dose of dopamine (omission error)
Mannitol 25 mg IV QID was prescribed as a maintenance dose for 50 kg patient with increase ICP, instead of 25 g IV QID (wrong dose) Captopril was prescribed for a patient with hyperkalemia (k:5.3) to control hypertension (wrong indication) Hydrochlorothiazide was abbreviated as Hct (wrong abbreviation)

## Discussion

In the present study, prescribing errors were investigated in the medical ICU of TASH. The higher prevalence of these errors (40.7 %) in the prescribing process indicated a need for improvement in ordering stage of the medication use process. None of the errors identified were fatal, but approximately one-third was assessed as being potentially significant or serious.

The rate of prescribing errors found in this study (40.7 %) is a relatively low frequency compared to the results of a recent study done in medical ICU of Jimma University Specialized Hospital (JUSH) involving 69 patients with error rate of 52.5 % [4]. Whereas, comparing this result with those from a study by Bates et al. [16], using observational method in an adult patient population, our study had a higher rate of medication errors (40 errors/100 orders in our study versus 5.3/100 orders). In an observational study conducted in Moroccan medical ICU, the error rate was 10/100 orders [17], Iran (35.1 %) [2] and the study in Brazil showed 7.47 % error rate [10], the rate in our study is still higher. Whereas the error prevalence in the current study is comparable with the study in Denmark (41 %) [15].

The systematic review of ME incidence in different ICU types [8], have found a wide variation in reported rates of MEs. The difference could be due to differences in definitions of errors, methods used to detect errors, level and type of ICUs, level of prescribing physicians and availability of facilities for patient care. However, the higher frequency of errors in the ICU of TASH even after excluding errors related with illegible hand writing, indications without drug, lack of authentication, and dosage form might be related to absence of a pharmacist in the health care team, absence of a closed-loop electronic prescribing mechanism, and lack of required medical facilities in the ICU.

Concerning the error types, the most common types of medication prescribing errors were omission of dose/frequency/route (42.89 %), the wrong combination of drugs (28.13 %) of which 57 (15.88 %) were drug interactions and 44 (12.26 %) duplication errors, wrong abbreviations (13.37 %), dose error [8.36 %; over dose (3.89 %) and under dose (4.46 %)], wrong frequency (5.01 %) and wrong indication (2.23 %). The present study indicated that, omission and combination errors account for about 71 % of the total error types. This result is higher than the finding of the study from Jimma (49.5 %) [4]. High number of omission and combination errors might be attributed to documentation problem, high turnover of prescription episodes and absence of any system to support prescribing physicians, who usually rely on their memory. According to the current study, the three most common categories of drugs encountered in prescribing errors were cardiovascular drugs (33.90 %), antimicrobials (20.49 %), and gastro-intestinal drugs (18.78 %). High incidence of error in these drug classes might be due to frequent prescription of these drugs. This finding was different from what was reported from UK, where cardiovascular 24.2 %, followed by blood and nutrition 19.9 %, CNS 16.1 %, infection 12.5 %, anesthesia 9.4 %, gastro-intestinal 5.3 % and endocrine agents 4.1 % [17]. The frequency of errors associated with gastro-intestinal drugs (20.49 %) was quite high in this study unlike the UK’s finding (5.3 %) and there were no errors recorded in relation to wrong route of administration. The difference in the error rate between drug classes might be attributed to differences in the types of cases admitted to the ICU and co-morbid conditions in the patients.

In the UK study percentage of drugs incorrectly prescribed was 9.4 % [17] unlike our study which is 21.5 %. This variation is probably due to the difference in the study set up where, the UK’s study is multi-centered. But, our result is comparable with the Brazilian study (20.2 %) [10]. The top five drugs with high error rate are different from those of UK’s study. The five most common incorrect prescriptions were for cimetidine 48 (11.71 %), clopidogrel 41 (10 %), omeprazole 20 (4.88 %), dopamine 19 (4.63 %) and tramadol 18 (4.39 %). More than one-third of the total error occurred in these five drugs (35.6 %).

The literature review revealed that there are various risk factors for high medication error rate in ICU patients like, age, gender, number of co-morbidities, number of drugs prescribed, state of the patient at admission and the length of stay in ICU [6]. Our finding is in concurrence with other studies in which the error rate is significantly associated with poly-pharmacy and age. In contrary to other studies, number of co-morbidity, length of stay in ICU and state of patient at admission were not significantly associated. This might be attributed to different confounding factors.

Our literature review highlighted that potential strategies to prevent medication errors in the ICU are focused on seven prevention strategies: eliminating extended physician work schedules, computerizing physician order entry, implementing support systems for clinical decisions, computerizing intravenous devices, and having pharmacists participate in the ICU, reconciling medications and standardizing medications [7].

The practical approach is to recognize that errors are a reality of medicine and that all health care providers have a responsibility to ensure patient safety and to use caution in promoting interventions. Improved medication safety may be accomplished by optimizing the safety of the medication process, eliminating situational risk factors and adopting strategies to intercept errors and mitigate their consequences.

Finally, it should be noted that errors during prescription writing process due to documentation problem might have been intercepted by prescribing physician with informal/oral communication with the nurse. The use of guidelines rather than clinical opinions to determine error must also be noted.

This study has certain limitations; it was a retrospective study and suffers from all shortcomings of retrospective study, the scope of the study was limited to prescribing errors, the study hospital was a teaching hospital and single-centered study so that it will affect its generalize ability to other general hospitals and the scope of the study was limited to prescribing errors and didn’t assess other components of medication errors, so the finding should be interpreted with caution.

## Conclusions

The present study establishes that prescribing errors were highly prevalent in the ICU of TASH. The errors reported in this study clearly show that there are multiple causes for prescribing errors in the ICU of TASH. Approximately one-third of the errors were potentially significant. Omission errors are the most common followed by wrong combination errors. Cardiovascular drugs were the classes with high error rate followed by antimicrobials. Age and poly-pharmacy were the strongest predictors of prescribing error. These results can be used to improve quality of health care delivery. Hospital managers should strive to create better awareness about the possibility of medication errors at the prescribing phase among health care professionals. Further prospective study involving other components of medication error is also recommended.

## Abbreviations

ICU: intensive care unit
ME: medication errors
TASH: Tikur Anbessa Specialized Hospital
